# The Contribution of Individual, Social and Work Characteristics to Employee Mental Health in a Coal Mining Industry Population

**DOI:** 10.1371/journal.pone.0168445

**Published:** 2017-01-03

**Authors:** Robyn Considine, Ross Tynan, Carole James, John Wiggers, Terry Lewin, Kerry Inder, David Perkins, Tonelle Handley, Brian Kelly

**Affiliations:** 1 School of Medicine and Public Health, University of Newcastle, Callaghan, New South Wales, Australia; 2 Hunter Institute of Mental Health, Hunter New England Local Health District, Newcastle, New South Wales, Australia; 3 NHMRC Centre for Research Excellence in Mental Health and Substance Use, University of New South Wales, Sydney, New South Wales, Australia; 4 Centre for Resources Health and Safety, Faculty of Health and Medicine, University of Newcastle, Callaghan, New South Wales, Australia; 5 Hunter New England Population Health, Hunter New England Local Health District, Wallsend, New South Wales, Australia; 6 Hunter Medical Research Institute, New Lambton, New South Wales, Australia; 7 Priority Research Centre in Brain and Mental Health, University of Newcastle, Callaghan, New South Wales, Australia; 8 School of Nursing and Midwifery, University of Newcastle, Callaghan, New South Wales, Australia; 9 Centre for Rural and Remote Mental Health, University of Newcastle, Orange, New South Wales, Australia; Ruhr-Universitat Bochum, GERMANY

## Abstract

**Background:**

Evidence regarding the extent of mental health problems and the associated characteristics within an employee population is necessary to inform appropriate and tailored workplace mental health programs. Mental health within male dominated industries (such as mining) has received recent public attention, chiefly through observations regarding suicide in such populations in Australia and internationally. Currently there is limited empirical evidence regarding the mental health needs in the mining industry as an exemplar of a male dominated workforce, and the relative contribution to such problems of individual, socio-economic and workplace factors. This study aimed to investigate the mental health and associated characteristics among employees in the Australian coal mining industry with a specific focus on identifying modifiable work characteristics.

**Methods:**

A cross-sectional study was conducted among employees (n = 1457) across eight coal mines stratified by key mine characteristics (state, mine type and employee commute arrangements). Participants completed measures of psychological distress (K10+) and key variables across four categories (socio-demographic characteristics, health history, current health behaviours, work attitudes and characteristics).

**Results:**

Psychological distress levels within this sample were significantly higher in comparison with a community sample of employed Australians. The following factors contributed significantly to levels of psychological distress using hierarchical linear regression analysis: lower social networks; a past history of depression, anxiety or drug/alcohol problems; high recent alcohol use; work role (managers) and a set of work characteristics (level of satisfaction with work, financial factors and job insecurity; perception of lower workplace support for people with mental health problems.

**Conclusion:**

This is the first study to examine the characteristics associated with mental health problems in the Australian coal mining industry. The findings indicate the salience of mental health needs in this population, and the associated interplay of personal, social and work characteristics. The work characteristics associated with psychological distress are modifiable and can guide an industry response, as well as help inform the understanding of the role of workplace factors in mental health problems in a male dominated workforce more generally.

## 1 Introduction

Internationally it is estimated that 20% of the population experience common mental disorders in any 12 month period [1]. Mental disorders are major contributors to the disease burden globally [1–3] particularly mood and anxiety disorders. In 2010 the global cost of mental disorders was estimated at $US2.5 trillion, with two-thirds of the total cost attributed to indirect costs such as lost productivity and income [4]. In Australia, during 2013–14, over $AU8 billion was estimated to be spent on mental health-related services [5]. Hilton et al [6] reported lost productivity of $AU5.9 billion annually associated with psychological distress.

Workplaces are important sites for addressing mental health problems for several reasons. First, mental health problems peak in working age, and occur across all educational, income levels and employment categories [1, 7–10]. Second, as a result of the significant time spent at work, workplaces provide an ideal site for intervention. Evidence indicates that workplace interventions can prevent mental health problems, and assist recovery for those with mental illness [11, 12]. As the majority of people experiencing mental illnesses receive no treatment [13], the workplace also provides an opportunity to encourage appropriate help-seeking particularly through combatting stigma. Third, the traditional focus on health protection and in particular safety in workplaces has evolved to recognise the potential impact of mental health problems to workplace health and safety practices and policy [14, 15]. Lastly, the growing recognition of the impact of mental illness on absenteeism, presenteeism, productivity and safety has elevated the organisational imperative to address this need [16–18].

Understanding the extent of mental health problems and the associated characteristics within an employee population, can guide appropriate and tailored workplace responses to promoting mental health and preventing mental illness. Community and workplace specific studies indicate that certain socio-demographic factors are significantly associated with mental health problems [19, 20]. Age, gender, current and past health problems, and family and social relationships have been associated with mental health problems [8, 19, 21]. While employment in a supportive organisational culture may be protective [8], other workplace factors such as long working hours [22, 23], regular overtime [24], high job demands with low levels of participation in decision making [25], job insecurity [26–28] and bullying, violence and discrimination in the workplace [29, 30] have been associated with an increased risk of mental health problems.

While the prevalence of mental health problems varies across occupations, there is limited empirical evidence about industry-related differences [22, 31]. Internationally, studies of occupation and mental health have frequently relied on general community-based surveys with comparisons across broad industry and occupational categories [10, 32]. While these might provide indicators of occupation-related risks, these studies are not able to inform more fine-grained understanding of the role of workplace factors in mental health. In a recent meta-analysis Battams et al [33] identified only nineteen studies between 1990 and 2012 which examined mental health problems and associated risk factors in male dominated industries, and four of these were subsets of general population studies. The prevalence rates for anxiety and depression using multiple measures varied across industries, and blue-collar workers experienced higher levels compared with white collar workers [33]. Using the Hopkins Symptoms Checklist-25 (HSCL-25) Nielsen et al [34] found that 9% of offshore oil workers reported high levels of psychological distress. In an Australian report examining mental health problems in mining, psychological distress was higher in a convenience sample of fly-in fly-out workers [35] than the Australian community [8].

The need for industry specific studies of mental health to inform tailored interventions has been advocated by a number of authors [33, 36]. Mental health problems in the Australian mining industry have received substantial public attention in recent times, resulting in numerous public enquiries [37, 38] focussing on the potential impact of work characteristics on employees’ mental health and suicide. The limited empirical evidence regarding the association between mental health problems and work characteristics in the mining industry has been a common finding of these enquiries [37, 38]. Internationally studies have identified elevated rates of suicide in male-dominated industries, including mining and construction, especially among less skilled workers [39, 40].

Coal mining contributes significantly to the Australian economy and employs 2% of the national workforce [41]. In Australia, the mining workforce is characterised by the following features: predominantly male (85%) [42]; most aged between 25 and 45 years [42]; with a relatively high income especially those working under fly-in fly-out (FIFO) or drive-in drive-out (DIDO) arrangements [43, 44]. Mining operations are also commonly located in rural and remote Australia, and may have limited local availability of health and social services and social supports.

Informed by the elements of a workplace psychosocial climate model [45] this study aimed to investigate the mental health and relative contribution of work characteristics among employees in the Australian coal mining industry, after accounting for socio-demographic, health history, and current health behavioural characteristics.

## 2 Methods

Ethics approval for the study was provided by University of Newcastle Human Ethics Committee (Approval Number H-2013-0135).

### 2.1 Sample and recruitment

Coal mines were selected using a quota sampling approach to ensure a representative cross-section of the industry. This process involved stratification of the selection criteria, to ensure representative coverage of mining companies, geographical location (two Australian States: New South Wales [NSW] and Queensland [QLD]), the mine type [underground or open-cut], and the main types of employee commute arrangements [fly-in fly-out (FIFO) / drive-in drive-out (DIDO) or daily commute/local]. Recruitment of coal mines and participants occurred between December 2013 and March 2015.

#### 2.1.1 Coal mines

Health and safety managers at company level were approached to secure company consent and to provide the names and contact details of general managers at ten mine sites. The research team then liaised with general managers at the ten mines to obtain consent for their individual mine to participate. This involved a member of the research team visiting the mine to provide a comprehensive overview of the research.

#### 2.1.2 Participant recruitment

In the weeks prior to data collection, each participating mine was provided with a range of promotional materials to be placed in employee common areas to promote awareness of the project. All staff, including all permanent mine staff and subcontractors, currently working at the participating mines on the day of data collection were invited to participate in the study.

### 2.2 Data collection procedures

To accommodate the unique and specific logistical considerations of each site, data collection occurred during routinely scheduled employee training days. At sites where training days were unavailable, data collection occurred while participants were on shift, or during their pre-shift meetings. A member of the research team visited the site and provided all participants with a written information statement and a brief presentation outlining the purpose of the research. Participation involved completing a pen-and-paper survey that took approximately 15 minutes to finish, with the return of the survey considered implied consent. Participation rate was determined by calculating the number of people who completed the survey, relative to the total number of employees at each mine.

### 2.3 Measures

#### 2.3.1 Psychological distress—Kessler 10

The Kessler Psychological Distress Scale 10+ LM (K10+) [46] was used to measure participants’ current level of psychological distress. The scale involves 10 items that measure the frequency of various negative emotional states in the preceding four weeks (5-level response scale).

#### 2.3.2 Characteristics associated with mental health problems

In order to account for variables previously associated with mental health problems in workplace studies [8, 22, 25, 31, 47, 48] the characteristics associated with psychological distress included a series of conceptually related variables grouped into four separate categories (socio-demographic, health history, current health behaviours, work characteristics). The grouping of these characteristics reflected the theoretical interest in examining the association between psychological distress and work characteristics, after accounting for the effect of the participants’: (1) socio-demographic status; (2) health history; and (3) current health behaviours.

For socio-demographic characteristics participants provided information on their age, gender, dependent children, and the highest level of education received. Social network factors were also assessed using Berkman-Syme Social Network Index [49] (SNI) which calculates a score based on: the number and frequency of contact with close friends and family; the presence of a spouse or intimate partner; any religious/social participation; and any community group participation. Low SNI scores reflected few or infrequent contacts and low group participation.

For health history, participants reported any previous diagnosis of a mental illness (i.e. depression, anxiety or substance use disorder), and/or a chronic physical health condition (i.e. heart attack or angina, high blood pressure, high cholesterol, other heart disease, stroke, cancer, diabetes, migraine,). Responses were categorised as: (1) no history; or (2) previous history.

Current health behavioural characteristics reflected a set of current behaviours that affect health including smoking status (categorised as: never smoked; ex-smoker; currently smoke, but less often than daily; or current daily smoker) and the frequency of use of three separate categories of illicit drugs including (1) cannabis; (2) synthetic drugs (e.g. KRONIK, synthetic cannabis); or (3) other illicit drugs with frequency categorised as: have never tried it; used, but not in the last month; or used in the last month. Hazardous and/or harmful drinking was measured using the 10-item Alcohol Use Disorders Alcohol Use Disorders Identification Test (AUDIT) [50].

The work characteristics included in the survey were categorised into work factors and work attitude factors (Table 1).

**Table 1:** Categorisation of Work Characteristics.

Factors	How it was measured
***Work factors***	
Mine type	Open cut or underground mining
Commute type	Single item question regarding commute arrangements: long distance commute (FlFO or DIDO)); daily commute (those who travel to and from work each day)
Years working in mining	Single item question that determined length of time working in the industry
Time to camp *(for long distance commute participants only)*	A measure of the duration of time to reach the mine site camp from the participant’s home. For multivariate analysis, we used dummy coding for all daily commute employees, with each participant given the mean response score of long distance commute participants
Daily commute time *(for daily commute participants only)*	A measure of the time taken to drive to work each day. For multivariate analysis, we used dummy coding for all long distance commute participants, with each participant given the mean response score of daily commute participants
Employment category	A single-item question about the employees’ specific occupational role from a list including: manager; professional; technician or trade worker; machinery operator and driver/labourer; or administration/other
Employment status	Single item that determined if participants worked full-time or part-time
Principal employee vs contractor	Single-item question to identify participants employed by the mine (principal employee) or as a contractor
Shift type	Asked participants to indicate whether they commonly work on a rotating shift pattern (mixture of day/evening/night shifts) or a regular shift (fixed day, or fixed night shift)
Shift length	Number of hours of the participants most common shift
Proportion of days at work	Using the participant’s typical roster, the proportion of time at work was a ratio of the number of consecutive days at work and the number of consecutive days off work
***Work attitudes***	
Satisfaction with work	An aggregate score based on the average responses given to seven items scored on a 5-point scale ranging from ‘very dissatisfied’ to ‘very satisfied’. Items include satisfaction with: Your usual take home pay; Your work prospects; The people you work with; Physical work conditions; The way your section is run; The way your abilities are used; and The interest and skill involved in your job.
Concern about losing job	A single item measured on a 5-point scale that asked participants to rate their level of concern about losing their job. Scores ranged from 1: ‘not at all’ to 5: ‘extremely worried’.
Work in mining for financial reasons	Aggregate score based on average response to three items scored on a 5-point scale ranging from 1: ‘strongly disagree’ to 5: ‘strongly agree’. Items include: The pay is the main reason I work in coal; I have financial commitments that mean I have to continue to work in coal mining because of the salary levels; I would prefer to work in another job but can’t afford to leave because of my financial commitments.
Work in mining because I love the work, and the roster suits my family	Average response to two items scored on a 5-point scale ranging from 1: ‘strongly disagree’ to 5: ‘strongly agree’. Items include: I work in coal because I love the work; the roster schedule suits my family and me.
Perception of the mines commitment to mental health	Average response to five items scored on a 5-point scale ranging from 1: ‘strongly disagree’ to 5: ‘strongly agree’. Items include: This mine would be flexible in offering work adjustments to someone with a mental health problem; This mine provides education and training to supervisors and managers about mental health; The managers at this mine have a good understanding of mental health issues; The mine provides education to employees about mental health; Our workplace policies support the mental health of mine employees.
Job Content Questionnaire (JCQ) [25, 51]	Items (psychological demands = 4 items; decision authority 2 items; skill discretion = 5 items) from the JCQ were utilised to measure the job-strain ratio, which was calculated using the formula: job strain ratio = mean of psychological demand / (the mean of decision authority and skill discretion). Thus, participants with a ratio score of 1 indicate balance between psychological demands and decision control; ratio score above 1 indicate that psychological demands outweigh decision control; ratio score below 1 indicate decision control is greater than psychological demands).
Perceived control over work	The average response to the two items reflecting perceived control scored on a 5-point scale ranging from ‘None’ to ‘Complete control’. Items include: The specific shifts that you work; The specific start and finish times that you work.

### 2.4 Statistical analysis

Microsoft Excel and Statistical Package for the Social Sciences (SPSS Version 22.0; Chicago, IL, USA) were used for all data analysis. Descriptive analysis was used to report sample characteristics and prevalence rates for K10. For all descriptive analysis, chi-square analysis was used where appropriate.

#### 2.4.1 Psychological distress

K10+ item scores were summed to give a possible cumulative score range of 10 to 50, with total scores categorised into four strata including low (10–15), moderate (16–21), high (22–29) and very high (30–50).

An analysis was undertaken to compare the psychological distress of the study sample to employed participants in the Australian National Survey of Mental Health and Well-being (ANSMHWB) [19]. The employed sample for the ANSMHWB was 5,495 persons aged 16–85 years who were usual residents of private dwellings across Australia. In this sample 49.1% were male with the 62% aged between 25 and 54 years.

#### 2.4.2 Characteristics associated with psychological distress

Factors associated with psychological distress were examined using hierarchical linear regression, with a predetermined order of entry for associative variables. As a partial control for the number of statistical tests, the α criterion was set at *p <* 0.01.

Preliminary principal components analyses were conducted to guide the derivation of subscale scores for several of the work attitude questions (e.g. work satisfaction; reasons for working in mining; and perceived commitment to mental health). All questions with aggregate scores were based on factors with an eigenvalue above 1.00. For scales, analysis reflected conventional scoring as reported in the literature for Berkman-Syme [49] and AUDIT [52].

Differences between recruitment methods were investigated using the same hierarchical regression model, with the exception of type of mining, which was moved to the first level to account for differences in the composition of the workforce for both open cut and underground mines.

#### 2.4.3 Missing data

Participants’ data were included if they answered a minimum of 80% of the K10 questions with missing data imputed using the average of all other questions provided.

## 3 Results

### 3.1 Participating mines

Ten mine sites were approached in NSW and Queensland, both states containing the majority of coal mining enterprises in Australia. Eight sites agreed to participate in the study with the remaining two mines unable to allocate sufficient time during the data collection window. The participating sites consisted of five in NSW and three in QLD, and a combination of: mine types (three open-cut and five underground) and commute arrangements (five daily commute and three long distance commute) for the workforce.

### 3.2 Mine employees

A total of 1,457 participants were recruited across the eight mine sites. Of the five sites where data collection occurred during routinely scheduled training/utility days, 929 of 1550 eligible employees completed the survey (participation rate: 60%, ranging from 30% to 72% across the five mines). At the remaining three sites, data collection occurred either while participants were on shift, or during their pre-start inductions. Across the three sites, 528 of 2386 employees completed the survey (participation rate 22%, ranging from 18% to 30%).

### 3.3 Sample characteristics

Table 2 outlines key personal and workplace characteristics of the sample. The sample consisted mostly of male (86.9%) participants, in the 25–44 years age category (61.3%) who were currently either married or in a de facto relationship (79.1%).

**Table 2:** Overview of Sample Characteristics.

Personal Variables	*n* (%)	Workplace Variables	*n* (%)
***Sex***		***Mine type***	
Male	1266 (86.9)	Open cut	773 (53.1)
Female	181 (12.4)	Underground	684 (46.9)
***Age***		***Mine workers***	
<24	116 (8.0)	FIFO/DIDO	414 (28.4)
25–34	448 (30.7)	Local	1040 (71.4)
35–44	446 (30.6)	***Work schedule***	
45–54	331 (22.7)	A regular shift	697 (47.8)
55+	105 (7.2)	A rotating shift	716 (49.1)
***Relationship Status***		Other	24 (1.6)
Not Married or de facto	205 (14.1)	***Most common shift length***	
Married and/or de facto	1152 (79.1)	8 hours or less	190 (13.0)
Separated/Divorced/ Widowed	88 (6.0)	9–12 hours	761 (52.2)
***Dependent Children***		More than 12 hours	500 (34.3)
No	597 (41.0)	***Employment Category***	
Yes	860 (59.0)	Managers	68 (4.7)
***Education***		Professional	198 (13.6)
<Yr 10	36 (2.5)	Trades worker	494 (33.9)
Yr 10 or equivalent	262 (18.0)	Machinery Operator	607 (41.0)
Yr 12 or equivalent	174 (11.9)	Administration or Other	89 (6.1)
Trade/Apprenticeship	524 (36.0)	***Years working in Mining***	
Certificate/Diploma	249 (17.1)	2 years or less	264 (18.1)
University or Higher degree	204 (14.1)	3 to 10 years	633 (43.4)
		xsMore than 10 years	549 (37.7)

The majority (67.2%) reported education at trade or apprenticeship levels or higher. Regarding work characteristics, there was an even representation of open cut (53.1%) and underground (46.9%) mining, however, a larger proportion of the sample identified as locally based (71.4%) employees when compared with DIDO or FIFO (28.4%) commuters. The occupation category and gender profile of the mine sample closely correlated to the industry profile of employees in the states in which the study was conducted [42].

After controlling for type of mine, minor differences were observed according to recruitment methods. Participants recruited from pre-shift were more likely: to have a degree; be concerned about losing their job; and be a full-time employee (p < 0.01).

### 3.4 Psychological distress

**Fig 1:**
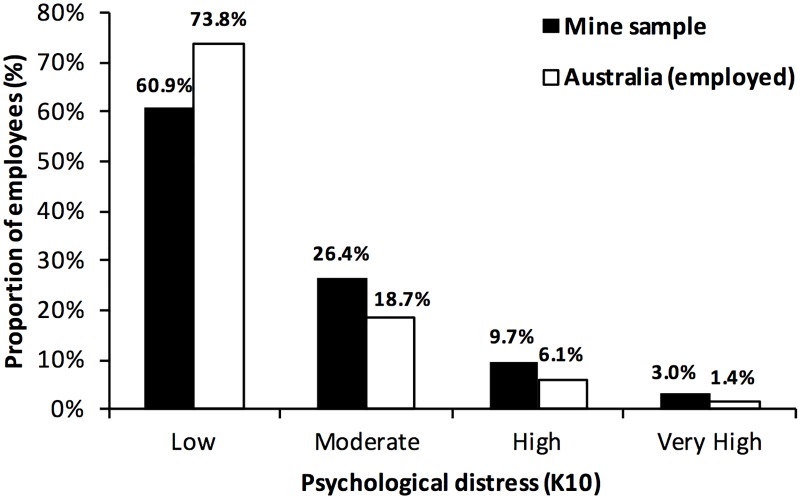
Psychological Distress (K10)—Comparison of the mine sample with age and gender weighted data of employed Australians (ANSMHWB).

Total K10+ scores detected in this sample were categorised as: low (60.9%), moderate (26.4%), high (9.7%) and very high (3.0%) (Fig 1). These rates were compared with age and gender weighted data of employed participants from the ANSMHWB [8]. After controlling for age and gender differences, the mining sample reported significantly higher rates of psychological distress than the comparable national data set χ2(3) = 97.81, p <0.001 [19].

### 3.5 Characteristics associated with psychological distress

Table 3 shows the results of a four-step hierarchical linear regression analysis including both univariate (i.e. Pearson correlations) and multivariate (i.e. standardised regression weights) associations between psychological distress and all independent variables investigated.

**Table 3:** Characteristics associated with psychological distress—results from a four-step hierarchical linear regression analysis.

Step/Variable	Psychological Distress (K10) score		
	Pearson Correlation	Adjusted *r*^2^	Standardised Regression Weights
**1. Socio-demographics characteristics**		**0.043**	
Age	-0.003		0.002
Gender (1. Male; 2. Female)	0.061		0.044
Dependent children (0. No; 1. Yes)	0.010		0.044
Education	0.042		0.043
TradeVsCert	0.055		0.050
Social Network Index (1-Low; 2-Medium; 3-Medium High; 4-High)	-0.188**		-0.188**
**2. Health history characteristics**		**0.164**	
Chronic physical condition (0. No; 1 At least one condition)	0.052		0.037
Depression (0, No; 1 Yes)	0.313**		0.196**
Anxiety (0, No; 1 Yes)	0.304**		0.180**
Drug or alcohol Problems (0, No; 1 Yes)	0.145**		0.091**
**3. Current health behavioural characteristics**		**0.189**	
AUDIT	0.160**		0.159**
Daily smoker (1, No; 2 Yes)	0.071*		0.009
Marijuana usage	0.066*		-0.022
Synthetic cannabis usage	0.061		0.028
Ecstasy, amphetamine or cocaine usage	0.087*		0.011
**4. Work characteristics**		**0.335**	
***Work Factors***			
Mine type (1-Open cut; 2-Underground)	0.046		0.047
Mine workers (1-FIFO/DIDO; 2-Local)	0.039		-0.018
Years working in Mining	0.069*		0.007
Time to Location (FIFO– 1 Low; 2 High)	0.010		-0.021
Time to Work (Local– 1 Low; 2 High)	0.066		0.023
Managers versus others	0.083*		0.071*
Professional Versus Technician and Machinery operators	0.054		0.022
Technicians versus machinery operators	0.007		0.014
Employment Status (1 Part-time; 2 Full-time)	0.060		0.031
Mine Employee Vs Contractor/subcontractor	-0.062		-0.042
Regular shift versus rotating shift (1 Regular; 2 Rotating)	-0.051		0.010
Most common shift length	0.014		0.058
Proportion of days at work	0.058		0.028
***Work attitude factors***			
Satisfaction with work	-0.404**		-0.233**
Concern about losing job	0.278**		0.159**
Work in mining for financial reasons	0.221**		0.105**
Work in mining because I love the work, and the roster suits my family	-0.233**		-0.042
Perception of mines commitment to mental health	-0.310**		-0.084*
JCQ—Perceived job demands v job control	0.046		-0.012
Perceived control over work	0.005		0.043

* *p* < 0.01;

** *p* <0.001

#### 3.5.1 Socio-demographic characteristics

Participant socio-demographic characteristics demonstrated a significant but minor contribution to psychological distress (4.3% of the total variance). Low social network scores were significantly associated with higher levels of psychological distress (β = -0.19, *p*<0.001).

#### 3.5.2 Health history characteristics

Personal health history characteristics accounted for an additional 12.1% of the total variance. Previous history of depression (β = 0.20, *p*<0.001), anxiety (β = 0.18, *p*<0.001) or drug or alcohol problems (β = 0.09, *p*<0.001) had a significant independent association with psychological distress. (*p* <0.001).

#### 3.5.3 Current health behavioural characteristics

Current health behavioural characteristics accounted for a further 2.5% of the total variance. Use of alcohol at risky or hazardous levels (scoring eight or above on the AUDIT) was significantly associated with higher levels of psychological distress (β = 0.16, *p*<0.001).

#### 3.5.4 Work characteristics

Work characteristics accounted for a further 14.6% of the total variance. Occupational group was significantly associated with managers reporting higher levels of psychological distress (β = 0.07, *p*<0.01). A significant independent association was detected between a number of work attitude factors and higher levels of psychological distress: overall dissatisfaction with work (β = -0.23, *p*<0.001); job insecurity (β = 0.16, *p*<0.001); working in mining for financial reasons (β = 0.11, *p*<0.001); and the perception that mines were not committed to the mental health of employees (β = -0.08, *p*<0.001).

## 4 Discussion

The current study aimed to investigate the mental health of employees of the Australian coal mining industry. In regards to overall levels of psychological distress, the study found a significantly greater proportion of coal mining employees with psychological distress scores at moderate to high levels, when compared to a national community sample of employed persons [8]. The levels of psychological distress observed are consistent with a previous study of FIFO/DIDO mine workers in Western Australia, [8, 35] which found that 36% of the sample reported total K10 scores above 20. The levels of psychological distress among this mining sample are also similar to those reported from the Australian Defence Force (ADF) Mental Health Prevalence and Wellbeing Study (12.9% high/very high distress) [53]. This is a comparable sample given its male dominated workforce, long working hours, and requirements to work away from home.

This study also aimed to examine the factors associated with psychological distress in this population, with a view to informing the development of appropriate workplace mental health strategies. As with mental health problems in the community, and consistent with other research in workplace settings, the factors associated with psychological distress were an interplay of personal, social and health characteristics and those associated with the workplace [31]. Participants with fewer social connections, a previous diagnosis of depression or anxiety, and those with problematic drinking behaviour; were at an increased risk of mental health problems.

Workplace characteristics provide potentially modifiable factors that may be a focus for interventions to reduce the risk of mental health problems [15, 45]. In the context of the current economic environment in mining with job reductions, it is not surprising that participants with high psychological distress were significantly more likely to report greater concern about losing their job. In Australia and internationally, job insecurity has frequently been associated with adverse health outcomes and in particular, increased levels of mental health problems [54–57]. While this context needs to be accounted for in interpreting the results, it also provides greater impetus to address the stressors and resultant mental health problems in the industry. The finding that working in mining for financial reasons was associated with higher levels of psychological distress and in particular that pay is the main reason for working in mining aligns with the concept of ‘golden handcuffs’. This concept refers to the use of high remuneration packages to support recruitment and retention which may result in employees becoming dependent on the high salary provided. This may contribute to psychological distress by influencing employees to stay in the job despite their preference to work elsewhere.

Workplace support from colleagues and in particular supervisors, has been identified as protective for mental health problems [26, 58]. Aligned with the findings of the Whitehall study [58], which found that high levels of social support in the workplace were associated with better mental health in employees, the results of the current study indicate that those who believed that their workplace was not committed to the mental health of its employees reported higher levels of psychological distress. Managers were most likely to report higher levels of psychological distress when compared with all other employment categories. Evidence about the association between employment categories and psychological distress is varied. In line with other studies [10, 59] work and industry conditions rather than the employment category alone are likely to be a more plausible explanation of the higher levels of psychological distress in managers in coal mining, particularly during periods of economic downturn in the industry.

The higher levels of psychological distress in this study were not explained by the oft-cited imbalance between job-demands and job control [25, 26, 60]. The impact on psychological distress was minimal for those who perceived the psychological job demands outweighed their decision authority or job control, thus having a high job-strain ratio. This absence of an association was surprising, but may reflect higher rewards (i.e. high remuneration) mitigating the impact of the imbalance on the employees mental health. Further and in contrast with commonly reported anecdotal evidence, there was no significant association between psychological distress and the type of commuting arrangements (FIFO or DIDO arrangements versus locally employed), shift length, or the type of mining (open cut versus underground mining For those who self-reported FIFO or DIDO arrangements in the current sample, 65.7% indicated that their travel time to the site was less than three hours. While still remote, this may be less than the average travel time of those who work in mining under FIFO arrangements and requires further research to examine whether commute distance, and associated sense of isolation from support networks is a contributing factor.

The finding that shift length was not associated with increased risk of psychological distress contrasts with other studies where long working hours and overtime have been associated with an increased risk for mental health problems [24, 61, 62]. One possible explanation for this finding may be that the majority of the sample (86.5%) worked more than 9 hours a day thus differentiation between shift lengths was not possible.

## 5 Limitations

This study provides the first empirical evidence regarding the extent of mental health problems in the Australian coal mining industry. However a number of areas of potential bias are noted. Firstly, this research used a cross-sectional design which does not allow temporal sequence to be determined and therefore causal associations cannot be made.

A second limitation was the use of two recruitment methods, which was necessary to meet logistical considerations of each site. Despite variation in participation rate according to the recruitment methods, there were no method related statistical differences in psychological distress. Furthermore, results of a hierarchical logistic regression showed that most of the independent variables assessed were not associated with recruitment method. One notable exception was concern about job security which was lower in training day participants, a factor that was also associated with psychological distress. Nevertheless, we found that the sample characteristics overall for both methods of recruitment were representative of the industry profile based on age, gender and employment category.

The data from the ANSMHWB, collected in 2007 was used for the comparison with the coal mining sample. Factors which impact on the health of the community may have changed in this time period. Since the ANSMHWB and in line with international evidence [56, 57], the global financial crisis and changes in commodity prices may have impacted on miners and their mental health through greater employment and financial insecurity. Such factors should be considered when interpreting comparisons with national survey data.

## 6 Conclusion

The findings from this study support the importance of a focus on mental health for the mining industry, as it is for all workplaces, and industries. Mental health problems are common among coal mining employees and are at least equivalent to comparable populations. The personal and social factors associated with psychological distress highlight opportunities for the industry to address and respond within a broad health and social context. Similarly the work characteristics associated with higher levels of psychological distress are potentially modifiable within industry business models. Thus multi-component interventions which address personal and social factors as well as work characteristics are likely to benefit individuals and the industry.
